# Adrenal Insufficiency Induced by Checkpoint Inhibitor Therapy in a Patient With Metastatic Acral Melanoma: A Case Report

**DOI:** 10.7759/cureus.91338

**Published:** 2025-08-31

**Authors:** Faith Oluwaseun Williams, Irene Katsaiti, Arianne Garcet, Uthra Rajendran, Srikanth Akunuri

**Affiliations:** 1 Acute Medicine, King's College Hospital National Health Service (NHS) Foundation Trust, London, GBR; 2 Internal Medicine, King's College Hospital National Health Service (NHS) Foundation Trust, London, GBR; 3 General Medicine, King's College Hospital National Health Service (NHS) Foundation Trust, London, GBR

**Keywords:** acth, adrenal, cortisol, hyponatremia, sodium

## Abstract

Immune checkpoint inhibitors (ICIs) have transformed the therapeutic landscape of advanced melanoma; however, their clinical benefit is tempered by a widening spectrum of immune-related adverse events (irAEs), including endocrine dysfunction. Among these, hypophysitis and adrenal insufficiency remain under-recognised, yet potentially life-threatening complications.

We describe a case of secondary adrenal insufficiency in a 78-year-old female with unresectable Stage IIIC acral melanoma treated with nivolumab and relatlimab. She presented with profound hyponatraemia, hypotension, and fatigue. Subsequent evaluation confirmed central adrenal insufficiency. Glucocorticoid replacement led to rapid clinical improvement. This case underscores the imperative for heightened clinical vigilance in patients receiving ICI therapy. Recognition and prompt intervention are crucial to reducing morbidity and optimising oncologic and endocrine outcomes, which could range from thyroid dysfunction and adrenal insufficiency to hypophysitis, as the case underscores the clinical complexity of diagnosing ICI-related endocrinopathies and highlights the need for high clinical vigilance and structured endocrine surveillance in patients undergoing immune checkpoint blockade.

## Introduction

Immune checkpoint inhibitors (ICIs) have transformed the management of advanced malignancies, particularly metastatic melanoma, through targeted disruption of key immunoregulatory pathways such as PD-1, PD-L1, and CTLA-4 [1,2], potentiating durable antitumour responses and becoming central to first-line treatment protocols. More recently, the addition of lymphocyte-activation gene 3 (LAG-3) inhibitors, such as relatlimab, in combination with PD-1 blockade, has demonstrated further improvement in clinical outcomes, enhancing effector T-cell activity while circumventing tumour immune escape.

However, the clinical efficacy of ICIs is counterbalanced by a growing spectrum of immune-related adverse events (irAEs), which may affect multiple organ systems. Endocrine irAEs occur in approximately 20-25% of patients receiving monotherapy and rise to over 40% with combination regimens [3]. Among these, thyroiditis and hypophysitis are most frequently reported, while adrenal insufficiency, both primary and secondary, remains rare but clinically significant. The incidence of adrenal insufficiency ranges from 0.7% to 4%, depending on the ICI regimen [4,5].

Importantly, these figures likely underestimate the true burden of disease. The presentation of ICI-induced adrenal insufficiency is often nonspecific, such as fatigue, hypotension, anorexia, and hyponatraemia, and easily misattributed to malignancy progression, infection, or systemic therapy effects. Moreover, isolated adrenocorticotropic hormone (ACTH) deficiency due to lymphocytic hypophysitis may not produce overt radiological changes, leading to further diagnostic delays [6,7]. Underrecognition and underreporting are compounded by the absence of routine endocrine screening in most oncology protocols.

The introduction of dual immune checkpoint blockade, particularly LAG-3/PD-1 inhibition, further intensifies immune activation and has been associated with an increased risk of multi-endocrine dysfunctions. Early reports suggest that polyendocrinopathies, including adrenalitis and isolated ACTH deficiency, may be more prevalent with these regimens, although the mechanisms remain incompletely understood [8,9].

Here, we report a case of secondary adrenal insufficiency in an elderly patient with metastatic acral melanoma receiving combination nivolumab and relatlimab.

## Case presentation

A 78-year-old woman with a history of Stage IIIC metastatic acral melanoma (BRAF wild-type) presented to the Medical Same Day Emergency Care (MSDEC) unit with new-onset lethargy and confusion. Her past medical history included surgical resection of lesions involving the right ankle and groin, in-transit metastases of the right lower limb, and nodal disease. She had been commenced on a combination immunotherapy with nivolumab and relatlimab on 18 June 2024 and had completed three cycles at the time of presentation.

Her past medical history was notable for hypertension, for which treatment had recently been discontinued due to normalisation of blood pressure, despite not taking antihypertensives. Regular medications included atorvastatin 40 mg once daily and loperamide, taken as required for diarrhoea.

She had been referred by her general practitioner (GP) following the incidental identification of hyponatraemia on routine blood tests. The patient reported a recent COVID-19 infection, accompanied by persistent diarrhoea lasting two to three weeks and subsequent anorexia. Loperamide had been prescribed in the community following unremarkable stool culture results. Additionally, she reported several recent episodes of syncope, and her family expressed concern regarding progressive forgetfulness and missed appointments, which they described as atypical for her.

Examination and investigations

On admission, the 78-year-old female presented with a low sodium level of 118 mmol/L (133-146 mmol/L) from the GP and a swollen right lower leg due to post-surgical lymphoedema, accompanied by mild ankle oedema, but without signs of hyperpigmentation. She was hypotensive, with a blood pressure of 88/54 mmHg, and had a low capillary blood glucose level of 3.4 mmol/L (4-11.1 mmol/L). Initial laboratory investigations revealed a serum osmolality of 240 mOsm/kg (280-295 mOsm/kg), a 9 am cortisol level of 47 nmol/L, and potassium at 4 mmol/L (3.5-5.5 mmol/L). Thyroid-stimulating hormone (TSH) was slightly elevated at 5.24 mIU/L (0.35-4.94 mIU/L). Urine studies showed an osmolality of 493 mOsm/kg and a urine sodium concentration of 32 mmol/L. Based on the clinical presentation and investigation results, a diagnosis of adrenal insufficiency secondary to ICIs was made by the endocrinologist.

**Figure 1:**
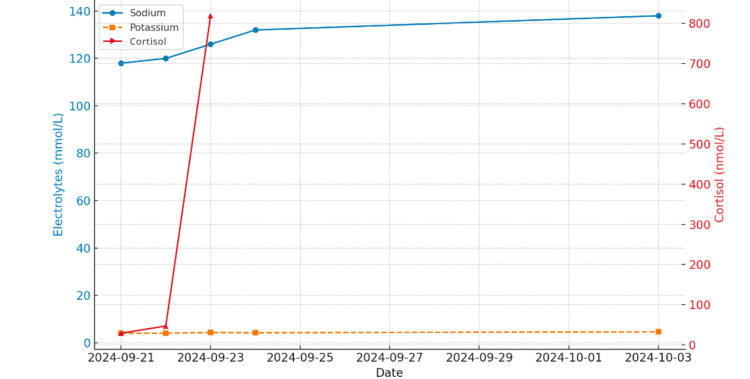
Trends in sodium, potassium and cortisol during admission The image is created by the author.

The patient’s serum sodium levels improved rapidly following initiation of hydrocortisone, while potassium remained within normal limits throughout, consistent with secondary adrenal insufficiency and preserved aldosterone function (Figure 1).

Management and treatment

The management and treatment of ICI-induced adrenal insufficiency requires immediate intervention to prevent adrenal crisis. Recent clinical guidelines recommend early recognition and pre-emptive management of adrenal insufficiency in patients receiving ICI therapy, as this strategy is associated with improved long-term outcomes and reduced morbidity [10].

Acute management was provided by administering intravenous hydrocortisone, beginning with a loading dose of 100 mg, followed by 50 mg every six hours. Simultaneously, an infusion of normal saline was initiated to correct dehydration and support sodium balance. Blood glucose levels were closely monitored, and hypoglycaemia was corrected as needed through the administration of glucose. These measures were essential to stabilise the patient and prevent further metabolic decompensation.

The transition to long-term therapy was initiated once the patient’s serum sodium level had improved to above 126 mmol/L and oral intake was considered sufficient. After two days in hospital, she was switched to an oral hydrocortisone dose of 20 mg in the morning and 10 mg in the evening. 

Patient education was a crucial aspect of management, particularly in preparation for ongoing self-care. The patient was counselled on the need for lifelong steroid replacement therapy and was instructed to adjust dosing during periods of illness or surgical stress. She was also advised to carry a steroid emergency kit containing hydrocortisone injections for use in the event of an adrenal crisis. Additionally, the patient was encouraged to wear a medical alert bracelet indicating her diagnosis to facilitate rapid intervention in emergency situations.

Follow-up and monitoring were arranged to assess recovery and screen for additional immune-related endocrinopathies. An endocrinology review was scheduled, and the oncology team was consulted to determine the feasibility of resuming ICI therapy, with the condition that close endocrine surveillance would be maintained, ensuring appropriate management of adrenal function and early detection of any emerging endocrine complications.

## Discussion

Signs and symptoms of adrenal insufficiency

**Figure 2:**
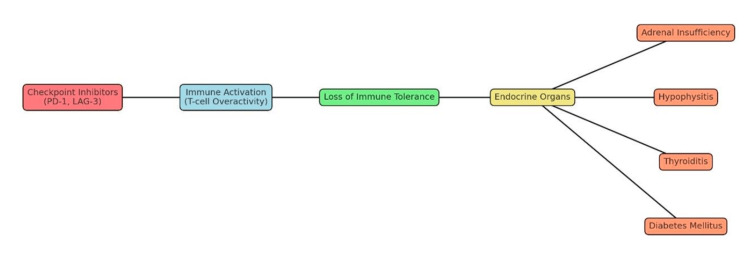
Pathways from checkpoint inhibitor therapy to endocrine immune-related adverse events The image is created by the author.

Adrenal insufficiency represents a clinically heterogeneous disorder, often characterised by insidious and non-specific symptomatology, which may complicate timely diagnosis. The clinical manifestations differ according to the underlying aetiology, with primary adrenal insufficiency typically associated with features such as hyperpigmentation and hyperkalaemia due to concomitant mineralocorticoid deficiency. In contrast, secondary adrenal insufficiency, resulting from hypothalamic-pituitary dysfunction, more commonly presents without these distinguishing features. Nevertheless, a shared symptom complex comprising fatigue, hypotension, anorexia, and hyponatraemia is observed in both entities (Figure 2). In the present case, the prominence of these non-specific but physiologically significant features highlights the necessity for heightened clinical vigilance, particularly in patients undergoing immune checkpoint inhibitor (ICI) therapy, where immune-mediated endocrine dysfunction may present subtly yet progress rapidly if unrecognised. Recognition of these symptoms is critical to prevent adrenal crisis, which can be life-threatening if left untreated.

Endocrine toxicity of PD-1/PD-L1 inhibitors: mechanisms leading to adrenal insufficiency and hyponatraemia

ICIs, particularly PD-1 and PD-L1 antagonists, have transformed oncological care, yet they are increasingly associated with immune-related endocrine toxicities, including adrenal insufficiency and resultant hyponatraemia. Understanding the underlying pathophysiological mechanisms is essential for timely diagnosis and management.

Programmed cell death protein 1 (PD-1) and its ligand (PD-L1) function as key immune checkpoint regulators that preserve peripheral immune tolerance by constraining excessive T-cell activation. Under normal physiological conditions, PD-1 interacts with PD-L1 on antigen-presenting cells to suppress overactive immune responses and prevent autoimmunity [3,4]. In oncological contexts, however, tumours exploit this pathway by overexpressing PD-L1, effectively deactivating cytotoxic T-cells and thereby evading immune surveillance and destruction [5,6].

Checkpoint inhibitors, including PD-1 inhibitors (e.g., nivolumab, pembrolizumab) and PD-L1 inhibitors (e.g., atezolizumab, durvalumab), restore immune surveillance by blocking the PD-1/PD-L1 interaction, thereby enhancing T-cell cytotoxicity and promoting anti-tumour responses. While this immune reactivation is therapeutically beneficial, it concurrently removes critical self-tolerance mechanisms and increases the risk of immune-related adverse events (irAEs) [7,8].

**Figure 3:**
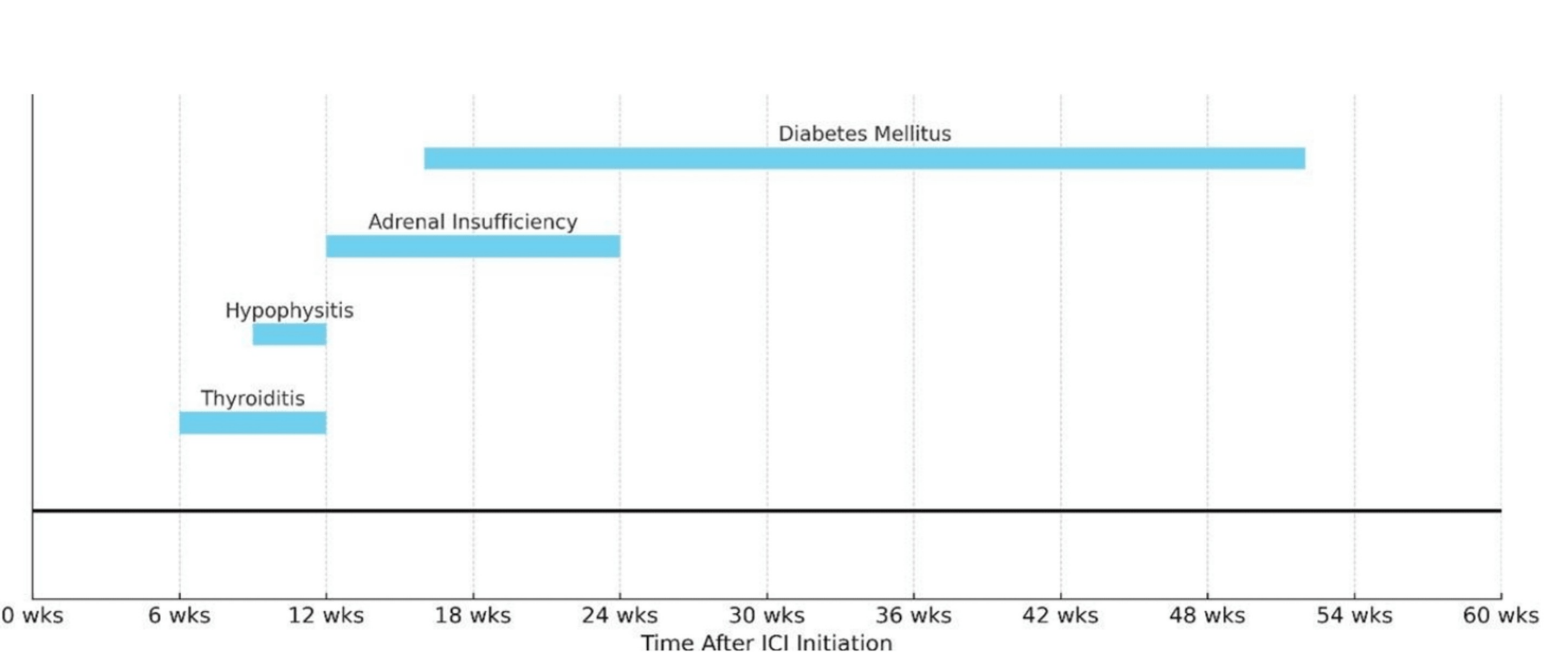
Typical timeline of onset of endocrine immune-related adverse events following initiation of checkpoint inhibitor therapy The image is created by the author.

The mechanisms by which PD-1/PD-L1 inhibitors induce adrenal insufficiency are multifactorial and predominantly immune-mediated. Autoimmune adrenalitis may arise through direct T-cell-mediated destruction of the adrenal cortex, frequently involving CD8+ T-cell infiltration and the generation of anti-adrenal antibodies targeting steroidogenic enzymes such as 21-hydroxylase [10,11]. In addition, PD-1 blockade may precipitate immune-mediated hypophysitis, leading to secondary adrenal insufficiency (SAI) due to ACTH deficiency. Unlike CTLA-4 inhibitors, which tend to cause global pituitary dysfunction, PD-1 inhibitors are more commonly associated with isolated ACTH deficiency, sparing other pituitary hormones [12,13]. Loss of peripheral tolerance also plays a pivotal role, as inhibition of the PD-1/PD-L1 axis enhances autoreactive T-cell activation and reduces immune restraint towards endocrine organs, including the adrenal glands, thyroid, and pancreas [14-16]. This dysregulation is further amplified by the overproduction of pro-inflammatory cytokines such as IFN-γ, IL-17, and TNF-α, which exacerbate tissue inflammation and contribute to both adrenalitis and pituitary dysfunction [17-20] (Figure 3).

From a clinical perspective, these immunopathological mechanisms account for the variable presentations of adrenal insufficiency in patients receiving PD-1/PD-L1 therapy, ranging from subtle manifestations such as fatigue and hyponatraemia to life-threatening adrenal crisis. Prompt recognition, endocrine assessment, and patient education regarding lifelong steroid replacement and emergency management are essential to mitigate morbidity and optimise outcomes in this scenario.

The temporal evolution of immune-related endocrine adverse events often follows a recognisable pattern. The sequence typically includes early-onset thyroiditis, followed by hypophysitis and, subsequently, adrenal insufficiency, each occurring within characteristic timeframes after the initiation of immune checkpoint inhibitor therapy. Notably, adrenal axis dysfunction tends to emerge within the first few weeks to months of treatment [21,22,13], a period during which heightened clinical vigilance is imperative. This pattern aligns with the presentation observed in our patient and underscores the importance of structured endocrine monitoring early in the immunotherapy course [23,24,11].

Lymphocyte-activation gene 3 (LAG-3) is an immune checkpoint receptor expressed on activated T-cells, regulatory T-cells (Tregs), and natural killer (NK) cells. It functions as a negative regulator of immune activation, playing a crucial role in preserving immune homeostasis and preventing excessive immune responses [25,26]. LAG-3 mediates its immunosuppressive effects through interaction with major histocompatibility complex (MHC) class II molecules, leading to attenuation of T-cell activation and diminished cytokine production [27,28].

Within the context of cancer immunotherapy, LAG-3 inhibitors such as relatlimab are employed to augment anti-tumour immunity by reversing T-cell exhaustion and restoring effector T-cell function [8]. The combination of LAG-3 inhibition with PD-1 blockade (e.g., relatlimab plus nivolumab) has demonstrated superior clinical efficacy in melanoma, sustaining T-cell activity against tumour cells while impeding immune escape mechanisms [29].

Despite these therapeutic benefits, LAG-3 inhibition is associated with an increased incidence of immune-related adverse events (irAEs), including endocrine dysfunction. One proposed mechanism is the enhancement of T-cell activation following LAG-3 blockade, which may precipitate autoreactive responses against endocrine organs such as the adrenal and pituitary glands [30]. Furthermore, the risk of polyendocrinopathy appears to be elevated when LAG-3 inhibitors are used in combination with PD-1 blockade, with reported occurrences of adrenalitis, hypophysitis, and thyroiditis [25,29]. Another contributory factor is the disruption of regulatory T-cell function, as LAG-3 expression on Tregs is integral to immune tolerance; its inhibition may reduce Treg-mediated suppression and thereby increase susceptibility to autoimmune-mediated endocrine injury [28,30].

A comparative analysis of endocrine toxicities associated with PD-1, PD-L1, and LAG-3 inhibitors indicates that the risk of adrenal insufficiency is markedly higher with combination regimens, particularly those incorporating LAG-3 blockade [31-33] (Table 1). Emerging evidence underscores that dual checkpoint inhibition significantly heightens the incidence of endocrinopathies relative to monotherapy, with adrenal insufficiency more frequently reported in patients receiving anti-PD-1/LAG-3 combinations than in those treated with anti-PD-1 monotherapy [30]. These observations highlight the necessity for early and systematic monitoring of adrenal function in patients receiving such immunotherapeutic protocols [8,29].

**Table 1:** Comparing PD-1, PD-L1, and LAG-3 inhibitors in endocrine toxicities

Feature	PD-1 Inhibitors (e.g., Nivolumab, Pembrolizumab)	PD-L1 Inhibitors (e.g., Atezolizumab, Durvalumab)	LAG-3 Inhibitors (e.g., Relatlimab)
T-cell Activation	Direct blockade of PD-1 on T-cells, stronger immune activation	Indirect effect via PD-L1 blockade on tumor and immune cells	LAG-3 blockade enhances T-cell function by preventing T-cell exhaustion
Risk of Endocrine irAEs	Higher risk due to stronger T-cell activation	Lower risk, but still present	Moderate risk, particularly when combined with PD-1 inhibitors
Common Endocrinopathies	Hypophysitis, adrenalitis, thyroiditis	Thyroiditis, diabetes, mild pituitary effects	Adrenal insufficiency, thyroid dysfunction, polyendocrinopathy
Prevalence of Adrenal Insufficiency	~1-3% in melanoma trials	<1% in most solid tumor trials	Exact prevalence unclear, but increased risk when used with PD-1 inhibitors

While future advancements may yield predictive biomarkers or risk stratification models, current clinical practice should rely on vigilance, interdisciplinary collaboration, and a readiness to question assumptions, lest the therapeutic promise of immunotherapy be compromised by delayed recognition of its endocrine repercussions.

## Conclusions

This case highlights the evolving landscape of endocrine pathology in the context of modern oncological therapies, specifically the nuanced presentation of secondary adrenal insufficiency induced by dual immune checkpoint inhibition. As immunotherapy becomes increasingly integral to the treatment of advanced malignancies, clinicians must develop a parallel fluency in recognising its endocrine consequences. The symptoms of adrenal insufficiency demand a refined diagnostic sensibility, especially as these manifestations often overlap with those of systemic illness, chemotherapy, or tumour burden itself. In patients receiving agents such as nivolumab and relatlimab, these symptoms warrant urgent hormonal assessment rather than being dismissed as non-specific.

More broadly, this case illustrates why structured endocrine surveillance is essential within immunotherapy care pathways. As combination checkpoint blockade becomes more prevalent, the incidence of immune-mediated polyendocrinopathy is increasing. Timely recognition, prompt initiation of glucocorticoid therapy, and long-term endocrine follow-up are crucial in reducing morbidity and ensuring that oncological benefits are not overshadowed.
